# Acceptable health and ageing: results of a cross-sectional study from Hungary

**DOI:** 10.1186/s12955-020-01568-w

**Published:** 2020-10-20

**Authors:** Márta Péntek, Job van Exel, László Gulácsi, Valentin Brodszky, Zsombor Zrubka, Petra Baji, Fanni Rencz, Werner B. F. Brouwer

**Affiliations:** 1 Department of Health Economics, Corvinus University of Budapest, Budapest, Hungary; 2 Health Economics Research Center, University Research and Innovation Center, Óbuda University, Budapest, Hungary; 3 Erasmus School of Health Policy & Management, Erasmus University Rotterdam, Bayle Building, Office J8-51, PO Box 1738, 3000 DR Rotterdam, The Netherlands; 4 Erasmus School of Economics, Erasmus University Rotterdam, Rotterdam, The Netherlands; 5 Premium Postdoctoral Research Programme, Hungarian Academy of Sciences, Budapest, Hungary

**Keywords:** Acceptability, Ageing, Health-related quality of life, EQ-5D-3L, Hungary, The Netherlands

## Abstract

**Background:**

We aimed to investigate the acceptability of imperfect health states in relation to age in Hungary and analyse its determinants. Results are contrasted to age-matched actual population health scores and to findings from a previous study in The Netherlands.

**Methods:**

A cross-sectional online survey was performed. The same survey questions were applied as in a previous study in The Netherlands in order to enable inter-country comparisons. The descriptive system of the EQ-5D-3L health status questionnaire was used to assess the acceptability of moderate and severe health problems at ages from 30 to 80 by 10-year age-groups. Descriptive statistics were performed and linear regression analysis was used to investigate the determinants of acceptability.

**Results:**

Altogether 9281 (female 32.8%) were involved with mean age 36.0 years and EQ-5D-3L index score of 0.852 (SD 0.177). Acceptability of health problems increased with age, differed per health domain and with severity of the problems. Except for ‘Self-care’, moderate health problems were acceptable by the majority from age 70 and acceptability scores were lower than EQ-5D-3L population norms from that age. The lowest average acceptability age was found in the ‘Anxiety/depression’ and dimension the highest in the ‘Self-care’ dimension. Respondents’ age, current health, and lifestyle were significant determinants (R^2^: 0.041–0.130). With a few minor exceptions in some health dimensions, acceptability levels and patterns were strikingly similar to the Dutch findings.

**Conclusion:**

In Hungary, acceptability of health problems increases with age and the majority found severe problems never acceptable. Views on acceptability of health problems seem to be fairly generalizable across European countries with different health and economic indicators.

## Background

Acceptability has become an increasingly important topic in healthcare. Patients’ preferences for and acceptability of different types of diagnostics, drug administration methods and disease management modes have been studied in various diseases as these can significantly influence patients’ agreement and compliance with, as well as uptake of and participation in care [1–3]. Acceptability of health states and health changes, for instance in relation to progression of age, have been investigated less frequently, although these may also be relevant in the context of individual and societal decisions [4–6].

In general, health of most individuals is not ‘perfect’ (i.e. most individuals have a certain degree of impairment in some dimensions of health) [7], and deteriorates with age [8]. Individuals may perceive some health problems and imperfect health states as ‘normal’, and experiencing increasing problems and poorer health states as a natural part of the ageing process [9, 10]. This may cause certain imperfect health states to be considered ‘acceptable’. The number of imperfect health states considered acceptable may increase with age, both from an individual and a societal viewpoint. As an example, having some problems with mobility may be seen as unacceptable at the age of 30, but be considered fully acceptable at the age of 90. Whether or not something is seen as acceptable, likely depends on the health domain in which problems occur (e.g. pain may be judged differently than mobility), the level of problems, and the total health profile [5]. Moreover, perceptions of acceptability may be related to how healthy people are at different ages, on average, as individuals may compare themselves to others in evaluating their health [6, 11].

Experiencing acceptable health problems, or being in an acceptable, yet imperfect health state, may be associated with a lower probability of seeking health care or accepting treatments at the individual level. Moreover, at the societal level, priority may be given to treatments that are aimed at patients in ‘unacceptable’ health states, that is, below some threshold of acceptability [6]. Hence, knowledge on which health problems and states people consider to be acceptable at different ages can be informative in different contexts. Knowledge on this issue is, however, scarce.

Acceptability of health problems at different ages was investigated in two empirical studies in The Netherlands [4, 5]. Results of a first web-based survey in a relatively small convenience sample suggested that people often consider less than perfect health states acceptable, especially those involving moderate health problems. The acceptability of health problems varied per health domain and increased with the age of the person experiencing the problems [4]. Recently, this study was repeated and expanded in a larger sample, aged 18–65 years, that was representative for the Dutch general public [5]. Results of this study confirmed the previous findings, demonstrated the relevance of health profiles and identified some determinants of acceptability (like age of death of next of kin and having a healthy diet) [5].

The fact that people hold age-specific reference points for acceptable health can have significant implications for health care. Shared decision making may be enabled by integrating issues of acceptability of health problems in the communication between clinicians and patients [12]. A better understanding of patients’ views regarding the acceptability of health problems can modify treatment goals, may influence the evaluation of health gains and potentially patients’ compliance. Moreover, health gains above and below the acceptability level might be valued differently and receive different priority in health policy [4–6].

Despite its relevance and potential importance, evidence on age-dependent acceptability of health problems is still scarce and not available for most countries [13, 14]. One interesting question is the generizability of the Dutch findings to other populations. Especially since life expectancy as well as health expectancy differs between countries, one might expect inter-country variation in the evaluation of the acceptability of health problems in relation to age [15]. While health deteriorates with age in all societies, the moments and degrees of decline as well as the domains of health affected may vary significantly across countries, which could affect views on acceptability of health states.

In this paper, we investigate the acceptability of imperfect health states in relation to age in Hungary. Life expectancy at birth in Hungary is about 6 years lower than in The Netherlands. Moreover, health surveys reported better health status of the Dutch population than the Hungarian population, especially for ages 65 and over [8]. In addition, the quality of and access to health and social care services, as well as the cultural and socio-economic context, differs between the two countries. All these aspects might influence the age-dependent acceptability of health problems. The comparison of two countries that differ significantly in health indicators, health and social care systems, as well as in their economic development level, can add valuable knowledge regarding the impact of non-personal factors on acceptability of less than perfect health.

Hence, in this paper, we aim to assess the acceptability of imperfect health states in relation to age in Hungary. Since the same survey questions are used as in previous studies in The Netherlands [4, 5], we also discuss the inter-country differences and highlight the relationship between acceptability levels and the population norms of health in the two countries.

## Materials and methods

### Study design and participants

This study was part of a large survey, details of the study have been published elsewhere [16]. Briefly, an online survey (year 2008) was conducted in collaboration with and on the surface of a Hungarian web journal (‘Index’). Participation was voluntary and anonymous. No remuneration was offered to participants.

### Questionnaire

We used the set of questions used in the Dutch studies translated into Hungarian [4]. Moreover, respondents were asked about basic socio-demographic and relevant health variables, as well as about their subjective life expectancy (the age they expected to live, expressed in years) [5, 17]. Health status of the participants was assessed by the EQ-5D-3L questionnaire [18]. The descriptive part of the EQ-5D-3L covers five health domains (‘Mobility’, ‘Self-care’, ‘Usual activities’, ‘Pain/discomfort’ and ‘Anxiety/depression’) and respondents are asked to indicate their current health in each domain by choosing between three levels of responses (1: no problems, 2: some/moderate problems, 3: unable/extreme problems). Altogether 243 different health states can be described based on the answers. Utility values (i.e. EQ-5D-3L index scores) can be attached to each health state description obtained, reflecting social preferences for that specific health status on a scale from 0 (equal to being dead) to 1 (being in perfect health) with negatives scores refering to health states that are considered worse than death. We used the EQ-5D-3L utility value set (also called tariffs) of the United Kingdom—UK (range − 0.549 to 1.0) due to lack of country-specific tariffs in Hungary [19, 20]. The second part is a vertical visual analogue scale (EQ VAS) ranging from 0 (worst imaginable health state) to 100 (best imaginable health state). Participants were asked to indicate their current health state by marking the relevant point on the EQ VAS.

### Assessment of acceptability of health problems

Participants were asked to indicate which level of health problems they considered to be acceptable from ages 30, 40, 50, 60, 70 and 80 years and onwards or ‘never’ for different domains of health [5]. The descriptive system of the EQ-5D-3L questionnaire was used to describe health states and severity levels (see Additional file 1). The rate of responses was calculated for each age and for the ‘never’ choice. The average ages of acceptability for moderate and severe problem levels in each health domain were calculated using only the answers of respondents who did not indicate ‘never’.

### Acceptable health curve (AHC)

Acceptable levels of health expressed as EQ-5D-3L index scores were computed for ages 30, 40, 50, 60, 70 and 80 in two ways [5]. In the first approach (that we label ‘aggregate’ method), respondents’ answers on each single health domain for one age category were simply combined and the respective EQ-5D-3L index score was calculated. For instance, if someone indicated that some problems in ‘Mobility’, ‘Pain/discomfort’ and ‘Anxiety/depression’ would be acceptable from age 70 onwards, while indicating that no problems were acceptable at this age in the other two domains, the health state described as ‘21122’ would be considered as acceptable from age 70. Based on these index scores an acceptable health curve (AHC) was constructed, defined by the sample’s average acceptable EQ-5D-3L index score at each age (hereinafter: acceptable health curve—aggregate, AHC_AGGREGATE)_. In our second approach we [5] only let the worst acceptable health problem of the five domains determine the age of acceptability. For this purpose, the most severe problem was determined by the utility score of each level in each domain, not by the level itself. The AHC was constructed again based on the sample’s average scores (AHC_WORST)_. For instance, a health state that is described in the AHC_AGGREGATE_ calculation as ‘11223’ (some problems in ‘Self-care’ and moderate ‘Pain/discomfort’, severe problems in ‘Anxiety/depression’) would be considered as a health state of ‘11113’ in the AHC_WORST_ version. This is the most restrictive way of combining the responses obtained from respondents.

We compared the average acceptable health state values (AHC_AGGREGATE_ scores) of participants who believed to be alive at the age for which acceptable health states were asked (‘survivors’) with those of respondents who did not expect to reach that particular age (‘non-survivors’).

### Statistics

IBM SPSS Statistics 22 was used for the analyses. Besides descriptive statistics, we used linear regression analysis to investigate factors associated with acceptable health states (EQ-5D-3L index scores) by 10-year age groups, from age 30 to 80. Variables for age, gender, income level, employment, own health status, age of death of next of kin, and overestimation of life expectancy (where the substraction of age and gender matched statistical life expectancy from the subjective life expectany resulted higher than zero year) were considered for the analysis, as well as dummies for healthy lifestyle and smoking. Descriptive comparisons to most recent findings in The Netherlands [5] were performed, focusing on the differences and similarities in trends of the results in the two countries (statistical tests to compare the results were hampered by the lack of available person-level data from the Dutsch study). The present study included questions for ages from 30 to 80, while in The Netherlands an age range from 40 to 90 was used [5]. Therefore, responses on ages 30 and 40 were grouped in the Hungarian sample, as well as responses for age 90 and ‘never’ in the Dutch study.

## Results

### Sample characteristics

Altogether 15,300 respondents were routed into the online survey. Uncompleted cases were filtered out and only participants aged 18–100 years, also answering the gender question, were considered for the analysis. People who indicated to expect to live up to an age lower than their current age were excluded from further analysis. 9399 participants remained in the sample after using these criteria. Upon further inspection, 118 individuals (male N = 70, 59%; mean age 39.3 SD = 13.3 years) were excluded because of inconsistent answers. Table 1 presents main characteristics of the resulting sample (N = 9281) with. Participants’ mean (SD) age of 36.04 (10.58) years, EQ-5D-3L index and EQ VAS scores of 0.852 (0.177; N = 9018) and 76.68 (SD 19.15; N = 9281), respectively.

**Table 1:** Main characteristics of the study sample and general population (GP) reference values from Hungary

Variable	Category	N	%	GP%^a^
Gender	Female	3048	32.8	53.2
	Male	6233	67.2	46.8
Age (years)	18–24	1068	11.5	15.2^b^
	25–34	3874	41.7	18.6
	35–44	2492	26.9	16.3
	45–54	1176	12.7	17.5
	55–64	583	6.3	14.1
	65–74	78	0.8	10.5
	75–84	8	0.1	6.5
	≥ 85	2	0.0	1.3
Marital status	Married/living together	5960	64.2	49.2
	Single	2752	29.7	29.0
	Divorced	497	5.4	9.4
	Widow	72	0.8	12.2
Highest educational level	Primary	47	0.5	27.8^c^
	Secondary	2396	25.8	43.0
	High school	2861	30.8	8.3
	University	3977	42.9	4.7
Employment status	Full-time job	7577	81.6	–
	Part-time job	399	4.3	–
	Pensioner	215	2.3	–
	Disability pensioner	64	0.7	–
	Student	696	7.5	–
	Housewife	330	3.6	–
Net income (€/month)	0–249	795	8.6	–
	250–400	1294	13.9	–
	401–560	1798	19.4	–
	561–900	2550	27.5	–
	901–2260	2213	23.8	–
	≥ 2261	629	6.8	–
	Missing data	2	0.0	
Smoking status (> 5 cigarettes/day)	Yes	1664	17.9	–
	No	7603	81.9	–
	Missing data	14	0.2	
Healthy lifestyle	Healthier than most others	3758	40.5	–
	Comparable to others	4419	47.6	–
	Less healthy than most others	1102	11.9	–
	Missing data	2	0.0	
Kins' age at death (years)	< 55	35	0.4	–
	55–65	347	3.7	–
	65–75	2303	24.8	–
	75–85	4782	51.5	–
	85–95	1754	18.9	–
	> 95	60	0.6	–

^**a**^Hungarian Central Statistical Office, Microcensus, year 2005

^b^Age group, 15–24 years

^c^The share of people with educational level lower than primary school is 16.2%

### Acceptability of health problems at specific ages

The distribution of responses is presented in Table 2. Only few respondents indicated that moderate health problems were already acceptable at ages 30 or 40. Age 60 appeared to be a life stage in which moderate health problems became acceptable for a large number of participants. A majority of the respondents considered severe problems in any domain to be ‘never’ acceptable. The lowest average acceptability age was found in the ‘Anxiety/depression’ dimension (Table 2).

**Table 2:** Acceptability of less than perfect health states beyond a specific age by adult individuals from the general population in Hungary (year 2008), % of respondents (N = 9281)

Health domain (EQ-5D descriptive system)	Severity of problems	Health problems are acceptable from age …. and onward, cumulative %							Acceptable from age^a^, mean (S.D.)
		30 yr	40 yr	50 yr	60 yr	70 yr	80 yr	Never	
Mobility	Moderate problems	0.4	1.2	7.5	29.9	69.9	93.9	6.1	68.4 (9.3)
	Confined to bed	0.1	0.1	0.2	0.6	5.6	29.2	70.8	77.8 (5.3)
Self-care	Moderate problems	0.1	0.2	0.6	4.1	29.5	80.0	20.0	75.7 (6.4)
	Severe problems	0.1	0.1	0.1	0.3	2.9	30.3	69.7	78.8 (4.4)
Usual activities	Moderate problems	0.3	0.8	3.8	19.3	60.8	93.6	6.4	70.9 (8.6)
	Severe problems	0.1	0.2	0.3	1.1	7.9	47.4	52.6	78.0 (5.2)
Pain/discomfort	Moderate	2.1	6.1	18.1	43.1	74.9	92.7	7.3	64.4 (11.9)
	Extreme	0.4	0.6	1.7	5.8	19.0	46.2	53.8	74.1 (8.8)
Anxiety/depression	Moderate	10.1	16.0	24.2	34.3	49.3	60.9	39.1	58.0 (17.3)
	Extreme	3.4	5.0	8.1	11.8	19.6	31.4	68.6	64.7 (16.7)
Total, %	None	89.4	82.2	68.1	42.0	12.7	1.9	1.9	NA
	At least one moderate	10.6	9.3	21.4	47.8	77.5	69.1	47.1	NA
	At least one severe	3.4	1.8	3.8	7.4	22.8	59.6	85.6	NA

^a^Average age at which these health problems are considered acceptable, as indicated by those respondents who did not indicate ‘Never’. NA = not applicable

### Acceptable health states at specific ages

**Fig. 1:**
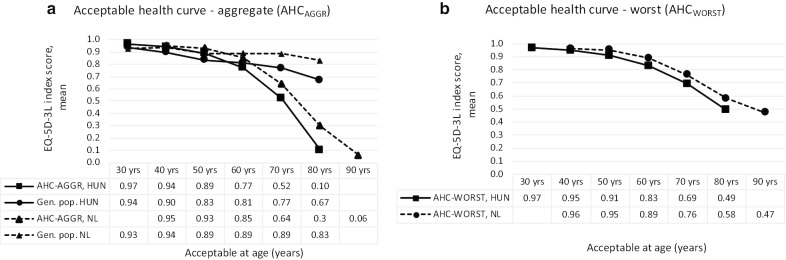
Average acceptable health states and actual health state scores in Hungary and in The Netherlands. *HUN* Hungary, *NL* The Netherlands, *Gen.pop* Actual health state score of the general population. *Notes* In the current study (Hungary) acceptable health problems were surveyed in 10-year intervals for ages from 30 to 80, whilst in The Netherlands (N = 1067) for ages from 40 to 90 [5]. AHC_AGGREGATE_ was calculated by the combination of single responses on 5 health domains, and for AHC_WORST_ only on 1 domain. The Dutch EQ-5D-3L utility tariffs were used in The Netherlands and the UK value set was used in Hungary. Average health state scores of the general populations (population norm) are presented for age groups 25–34, 35–44, 45–54, 55–64, 65–74 and 75 years and over [8]

Acceptable health curves are presented in Fig. 1a, b, respectively. Average AHC_AGGREGATE_ scores were lower than of the AHC_WORST_, which is a direct result of the calculation methods. However, the difference was especially meaningful for ages 70 and 80. Both AHC curves decreased with age, especially after age 60. Figure 1a also highlights the average health state of the general population of Hungary as measured by the EQ-5D-3L. Acceptable health states (AHC_AGGREGATE_) were similar to actual health state scores of the Hungarian general population up to age 60, but not for ages 70 and 80, where the AHC_AGGREGATE_ was lower than observed health states.

### Participants’ beliefs on longevity and acceptability of health problems

The average subjective life expectancy was higher than the gender and age-matched statistical life expectancy (mean 79.60, SD 10.83 vs. 73.53 SD 3.84 years). A majority of respondents (71.4%) overestimated their life expectancy (i.e. expected to live at least 1 year longer than their statistical life-expectancy). Mean AHC_AGGREGATE_ scores were significantly higher for each age in the ‘survivors’ subgroup than in the ‘non-survivors’ subgroup (*p* < 0.001) (Data not shown).

### Determinants of age-specific acceptability of health states

Results of linear regression analysis are presented in Table 3. It shows that those participants who overestimated their own life expectancy indicated higher scores. Those respondents who did not smoke and reported to live healthier than most others also showed higher acceptability scores. This suggests that respondents linked lifestyle factors to age-related acceptability of health problems, so that people who live healtier considered imperfect health states at later ages to be less acceptable. Next of kins’ age of death was associated with the acceptability of health problems from age 50 and beyond. Participants’ age and current health state were positively correlated with the acceptable health state scores, albeit with small coefficients. Some sociodemographic factors seemed to play a significant role as well. Students considered health problems at all ages to be more acceptable than other respondents did. Current higher net income was also associated with higher acceptable health state scores, but only for age 60 and over. However, we find important to note that R^2^ was rather low hence only few variants of acceptability were explained by the model inputs.

**Table 3:** Regression analysis

Variable	Acceptable health state …					
	At age 30	At age 40	At age 50	At age 60	At age 70	At age 80
*Constant*	0.856*	0.779*	0.518*	0.107**	− 0.447*	− *0.635*
Gender	–	–	–	–	–	0.035** (0.014; 0.056)
Age	0.001* (0.001; 0.002)	0.002* (0.001; 0.002)	0.002* (0.001; 0.002)	0.003* (0.003; 0.004)	0.005* (0.004; 0.006)	0.004* (0.003; 0.005)
Marital status	–	–	0.011*** (0.001; 0.020)	0.014*** (0.001; 0.027)	0.019*** (0.002; 0.037)	–
Highest educational level	–	–	0.006*** (0.000; 0.011)	0.014* (0.006; 0. 021)	0.012*** (0.001; 0.022)	–
*Employment status*						
Full time job	0.009*** (0.001; 0.018)	–	–	–	–	–
Part-time job	–	–	–	–	–	–
Pensioner	–	–	–	–	–	− 0.075*** (− 0.143; − 0.007)
Disability pensioner	–	–	–	–	–	–
Student	− 0.014*** (− 0.027; 0.000)	− 0.027* (− 0.040; − 0.014)	− 0.030** (− 0.049; − 0.012)	− 0.031*** (− 0.056; − 0.007)	− 0.050** (− 0.084; − 0.016)	− 0.066** (− 0.105; − 0.027)
Housewife	–	− 0.019*** (− 0.036; − 0.001)	− 0.032** (− 0.055; − 0.008)	–	–	–
Net income	–	–	–	< 0.000** (0.000; 0.000)	< 0.000** (0.000; 0.000)	0.000* (0.000; 0.000)
Current health status (EQ VAS)	0.000* (0.000; 0.001)	0.001* (0.000; 0.001)	0.001*	0.002* (0.001; 0.002)	0.002* (0.001; 0.002)	0.002* (0.001; 0.002)
Smoking status	− 0.019* (− 0.026; − 0.013)	− 0.021* (− 0.030; − 0.012)	− 0.027* (− 0.039; − 0.015)	− 0.043* (− 0.059; − 0.027)	− 0.049* (− 0.071; − 0.028)	− 0.065* (− 0.090; − 0.039)
Healthy lifestyle	–	0.012* (0.006; 0.017)	0.022* (0.015; 0.030)	0.033* (0.023; 0.042)	0.039* (0.026; 0.052)	0.032* 0.016; 0.047)
Kins' age at death	–	–	0.001* (0.001; 0.002)	0.003* (0.002; 0.003)	0.005* (0.004; 0.006)	0.004* (0.002; 0.005)
Overestimation of life expectancy	0.030* (0.025; 0.036)	0.048* (0.041; 0.056)	0.085* (0.075; 0.095)	0.130* (0.116; 0.143)	0.161* (0.143; 0.180)	0.107* (0.084; 0.130)
R^2^	0.041	0.058	0.093	0.130	0.121	0.059

Coding used for the analysis: Gender: female = 0, male = 1; Marital status: not married = 0, married = 1; Highest educational level: primary school = 1, secondary school = 2, college = 3, university = 4; Employment status related variables:: no = 0, yes = 1; Net monthly income: mean values of net income ranges presented in Table 1 were used for the analysis; Smoking status: no = 0, yes = 1; Healthy lifestyle: less healthy than most others = 1, comparable to most others = 2, healthier than most others = 3; Kins’ age at death: mean values of ranges presented in Table 1 were used for the analysis. Overestimation of life expectancy” takes the value of 1 if one overestimates his/her age and zero value otherwise

Beta coefficients (95% confidence intervals) are presented. Statistical significance of coefficients: *p < 0.001; **p < 0.01; ***p < 0.05

Method: stepwise, entry/removal criteria: 0.05/0.10

### Comparison of the results from Hungary and The Netherlands

**Fig. 2:**
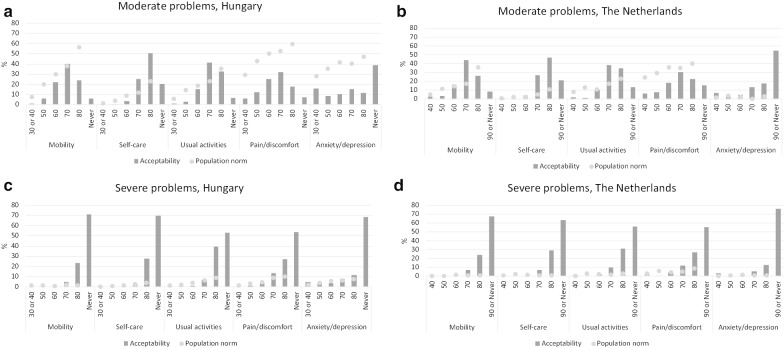
Acceptability rates of moderate and severe health problems in Hungary and The Netherlands and population norms*. *MO* mobility, *SC* self-care, *UA* usual activities, *PD* pain/discomfort, *AD* anxiety/depression. *Responses for ages 30 and 40 were summed in the Hungarian sample and responses for age 90 and Never were summed in the Dutch sample [5]. Source of population health status normative data: [8]

In general, the two countries show (strikingly) similar response patterns [5]. Regarding moderate health problems, problems in the ‘Pain/discomfort’ and ‘Anxiety/depression’ dimensions were indicated first as being acceptable in both countries, but with somewhat higher rates in Hungary (cumulative percent at age 50: ‘Pain/discomfort’ 18.1% vs. 13.8%; ‘Anxiety/depression’ 24.2% vs 9.3%, respectively). For age 60, the share of Hungarian respondents considering problems acceptable were higher in all domains except for ‘Self-care’, which scored very similarly in the two countries. Results again were more similar in both countries at ages 70 and 80 (see Fig. 2a, b).

Regarding severe health problems, only few respondents considered these to be acceptable in any domain of health before the age of 70. Similar trends were reported from The Netherlands [5], as it can be observed in Fig. 2c, d. For ‘Usual activities’ severe problems were considered somewhat more acceptable in Hungary at age 80. The proportions of respondents indicating that problems were ‘never’ acceptable were similar in the two countries. Another important similarity between the two samples was that 1.9% (Hungary) and 2.0% (The Netherlands) of respondents indicated ‘never’ for all health problems at all ages. This minority does not consider any health problem to be acceptable at any age.

When comparing the acceptability of moderate problems to the actual health status of the general public of the country, as also shown in Fig. 2, relevant differences can be observed between Hungary and The Netherlands. In the ‘Anxiety/depression’ dimension, the proportion of citizens in Hungary with moderate health problems is much higher than proportion of respondents labelling these problems as acceptable. In contrast, the two proportions are similar in The Netherlands in younger ages and acceptability exceeds the population norm rate at ages 70 and 80 there. In the ‘Pain/discomfort’ dimension the population norms were higher than the acceptability rates in both countries, however at a slightly diferent level. No major differences were observed between the two countries in relation to severe problems.

The patterns of the AHCs are very similar for the two countries (Fig. 1a, b). AHC scores were somewhat higher in The Netherlands than in Hungary, however, this is partly due to the higher utility scores of the Dutch value set compared to the UK value set [5, 19, 21]. Alternatively using the Dutch tariffs to calculate AHC_AGGREGATE_ for Hungary, importantly reduced the difference (see Additional file 2). The difference between AHC_AGGREGATE_ scores and respective population norms was very similar in both countries (Fig. 1a).

## Discussion

In this paper, we presented the results of a study on the acceptability of less than perfect health states at different ages in Hungary. Our results showed that certain health problems are acceptable for the Hungarian general public. The acceptability differed per health domain and with severity of the health problems, with severe problems in any domain considered to be unacceptable at any age by a majority of respondents. Moderate problems in ‘Anxiety/depression’ and ‘Pain/discomfort’ appeared to be acceptable earliest in life, and health problems were generally considered more acceptable in older ages. Respondents’ age, current health, and lifestyle were significant determinants of age-specific acceptability of health problems, although the influence of health and age appeared small. Those respondents who believed to be alive at a presented age (30–80) were also less likely to accept health problems at that age. This suggests that age-specific acceptable health problems and subjective life expectancy are related.

Our study has several strengths. This is the first study in Hungary (and also in the Central Eastern European region) to assess age-related acceptability of health problems. Our results can be used as country-specific reference points for acceptable health and provide some first insights into their determinants. Moreover, given that we used a similar methodology, we were able to compare the Hungarian findings to previous Dutch findings [5], although only in a descriptive manner. This comparison is however still interesting since both countries have quite different characteristics in terms of population health, health and social care systems and economic development levels. Considering that age-specific acceptable health problems were quite similar between these two fairly distinct countries suggests that views on acceptability of health problems may not differ substantially between (European) countries. We do note some differences as well. For instance, compared to the Netherlands, in Hungary a somewhat higher proportion of respondents indicated that moderate problems were acceptable under the age of 70. This difference mainly originated from differences in acceptability of moderate problems in the ‘Anxiety/depression’ dimension (see Fig. 2a, b). This may be related to the fact that the prevalence of problems in the ‘Anxiety/depression’ dimension among the Hungarian general population is much higher than that in The Netherlands (see Fig. 2), also in younger people [8]. One might also suspect both findings to be related to the wording of the validated Hungarian EQ-5D-3L questionnaire, in which ‘depression’ is translated as ‘lehangoltság’ (feeling down). However, high prevalences of problems in the ‘Anxiety/depression’ dimension were also reported for other Central and Eastern European countries [8, 22, 23]. This would support the validity of the Hungarian data. While this would also support that commonness of health problems may lead to higher acceptability of those problems, we emphasise that this is especially observed for anxiety and depression. For instance, we observed that a gap between the acceptability of health problems and the prevalence of actual problems in both the Dutch and Hungarian general population for the ‘Pain/discomfort’ dimension (see Fig. 2a, b). For that dimension, more problems were experienced than considered acceptable and higher prevalence seemingly did not translate into higher acceptability. On the other hand, the rate of citizens aged 64–75 reporting some problems in ‘Mobility’ was much higher in Hungary than in the Netherlands (37.7% vs. 17.1%), whilst acceptability rates at age 70 differed only slightly between the two countries (cumulative %: 69.1 vs 65.9). Overall, the association between prevalence of health problems of a population and the acceptability of these problems was limited. The acceptable health curves indicated that aggregated acceptability levels were close to population health status EQ-5D-3L index scores up to age 60 but diverge from age 70 onward in both countries, with aggregate acceptability profiles being below observed average health states.

Before highlighting some implications of our findings, we highlight some limitations of our study. First, our sample was not representative for the Hungarian population given our recruitment strategy and response rates. Young male respondents were overrepresented. This may have influenced our results and limits the comparability to the findings from The Netherlands in which a sample reasonably representative for the Dutch general public between the ages of 18 and 65 in terms of age, gender and education level were involved. Future studies are encouraged to include representative samples, also including respondents over 65 years old. Second, we had a limited set of ages for which we asked about acceptability of health problems. Including a broader range could provide important additional information (e.g. about how acceptable anxiety/depression would be in children and early adulthood) [24]. Third, we assumed that when a problem level was indicated to be acceptable at a certain age, it would be acceptable at older ages as well. This might be investigated further, for instance in relation to anxiety and depression. Fourth, the 3L version of the EQ-5D was used in both studies, not the more recent 5L. It could be interesting to see how people would respond to health problems described on a more sensitive instrument like the EQ-5D-5L [25]. Using other instruments that have different approach to health and well-being (e.g. ICECAP-A and ICECAP-O measures) could reveal additional new aspects [26]. Fifth, given the absence of Hungarian tariffs for the EQ-5D, we applied the UK tariffs to calculate AHC scores. Clearly, this UK data set need not necessarily reflect the preferences of the Hungarian population. Future studies are encouraged to use country specific tariffs, whenever available. Sixth, the gap between the AHC_AGGREGATE_ and AHC_WORST_ reflects the uncertainty about how to aggregate dimension-specific answers into a full health profile and EQ-5D-3L index scores. Future studies could include descriptions of full health states, in order to directly assess their acceptability. It was done in the Dutch study, but only for three profiles [5]. This provides information about the way in which people perceive combinations of health problems in several domains. Feasibility issues, also in relation to the more complex task and the high number of possible combinations, prevented us from doing this in this study. Using the EQ VAS to assess the acceptability of health states could be an interesting alternative approach. A recent pilot provided promising results regarding its applicability [27]. Seventh, patients with chronic diseases might have different views on the acceptability of health problems [12], hence further studies are suggested involving specific patient groups. Eigth, we emphasize that we considered only one aspect, namely the age, for the assessment of acceptability of health problems. Other relevant aspects could also be studied (for instance in relation to lifestyle), which may be relevant in the context of healthcare policies.

In terms of implications of our research, we highlight the following points. First, our results indicate that acceptability of health problems is common across countries, and increases with age. Moreover, severe problems in any health domain were acceptable for fewer individuals than moderate problems at all ages, and the great majority indicated that severe health problems were never acceptable. While general patterns between countries are similar, important differences (also for specific dimensions) can exist, which emphasises the value of country specific studies. Second, the fact that certain health problems may be seen as acceptable could have implications for how individuals perceive these problems (also at different ages) and whether they will seek care given the problems. Moreover, health care professionals may be more inclined to treat unacceptable problems than acceptable ones.

An interesting avenue for future research would be to see which problems medical professionals see as acceptable at different ages and whether this is associated with treatment choices. Moreover, at a societal level, priority may be given to treatments of those problems that are considered unacceptable. Whether such a way of setting priorities is in line with public preferences or normative ‘acceptable’ is another area for future research.

## Conclusions

Our results add useful knowledge to the recognition of what people consider acceptable health in different countries. Our study provided first results of acceptability of health problems in Hungary, could be compared to previous results from The Netherlands, and highlighted some interesting and relevant similarities and differences. A better understanding of acceptability of health problems at different ages may help to understand and explain health behaviours and treatment choices, and may ultimately be used to inform priority setting in medical and policy decision-making. Further research in this area, also in other countries and into the drivers of acceptability of health problems, remains warranted.

## Supplementary information

**Additional file 1**. Question to assess acceptability of health problems at specific ages, example.

**Additional file 2**. Acceptable health curve—aggregate (AHC_AGGR_) based on Dutch tariffs both in Hungary and The Netherlands.

## Data Availability

The datasets used and/or analysed during the current study are available from the corresponding author on reasonable request.

## Abbreviations

AHC: Acceptable health curve
AHC_AGGREGATE_: Acceptable health curve—aggregate
AHC_WORST_: Acceptable health curve—worst
EQ-5D-3L: Health status measure
EQ VAS: Health status thermometers (Visual Analogue Scale)
SD: Standard deviation
UK: United Kingdom
