# Supplementary material for: Acceptable health and ageing: results of a cross-sectional study from Hungary
Source: Health Qual Life Outcomes. 2020 Oct 20;18:346. doi: 10.1186/s12955-020-01568-w (PMC7574437; doi:10.1186/s12955-020-01568-w)
Supplement: Supplementary file 1 — Additional file 1. Question to assess acceptability of health problems at specific ages, example. [file 12955_2020_1568_MOSM1_ESM.docx]

**Title: Acceptable health and ageing: results of a cross-sectional study from Hungary**

**Journal: Health and Quality of Life Outcomes**

**Additional file 1: Question to assess acceptability of health problems at specific ages, example**

Can you indicate the age onward you consider the specified level of problems with ‘Mobility’ to be acceptable?

(Please indicate below the relevant age categories)

|  | Age category | | | | | | |
| --- | --- | --- | --- | --- | --- | --- | --- |
|  | From the age of 30 | From the age of 40 | From the age of 50 | From the age of 60 | From the age of 70 | From the age of 80 | Never |
| Some problems with walking about | □ | □ | □ | □ | □ | □ | □ |
| Confined to bed | □ | □ | □ | □ | □ | □ | □ |
