# Supplementary material for: Acceptable health and ageing: results of a cross-sectional study from Hungary
Source: Health Qual Life Outcomes. 2020 Oct 20;18:346. doi: 10.1186/s12955-020-01568-w (PMC7574437; doi:10.1186/s12955-020-01568-w)
Supplement: Supplementary file 2 — Additional file 2. Acceptable health curve—aggregate (AHCAGGR) based on Dutch tariffs both in Hungary and The Netherlands. [file 12955_2020_1568_MOSM2_ESM.docx]

**Title: Acceptable health and ageing: results of a cross-sectional study from Hungary**

**Journal: Journal: Health and Quality of Life Outcomes**

**Additional file 2. Acceptable health curve – aggregate (AHC_AGGR_) based on Dutch tariffs both in Hungary and The Netherlands**

HUN=Hungary, NL=The Netherlands, AHC=acceptable health curve

AHC_AGGREGATE_ was calculated by the combination of single responses on 5 health domains

Dutch tariffs, source: [21].
